# Arctic food and energy security at the crossroads

**DOI:** 10.1038/s43247-025-02122-6

**Published:** 2025-02-18

**Authors:** Adrian Unc, Majdi R. Abou Najm, Paul Eric Aspholm, Tirupati Bolisetti, Colleen Charles, Ranjan Datta, Trine Eggen, Belinda Flem, Getu Hailu, Eldbjørg Sofie Heimstad, Margot Hurlbert, Meriam Karlsson, Marius Korsnes, Arthur Nash, David Parsons, Radha Sivarajan Sajeevan, Narasinha Shurpali, Govert Valkenburg, Danielle Wilde, Bing Wu, Sandra F. Yanni, Debasmita Misra

**Affiliations:** 1 Memorial University of Newfoundland, School of Science and the Environment, Corner Brook, NL Canada; 2 McGill University, Department of Natural Resource Sciences, Sainte-Anne-de-Bellevue, QC Canada; 3 University of California-Davis, Department of Land, Air and Water Resources, Davis, CA USA; 4 Norwegian Institute of Bioeconomy Research, Division of Environment and Natural Resources; Ecosystems in the Barents region, Svanvik, Norway; 5 University of Windsor, Civil & Environmental Engineering, Windsor, ON Canada; 6 First Nations University of Canada, Indigenous Studies Faculty, Northern Campus, Prince Albert, SK Canada; 7 Mount Royal University, Department of Humanities, Calgary, AB Canada; 8 Norwegian Institute of Bioeconomy Research, Division of Environment and Natural Resources; Soil and Land Use, Ås, Norway; 9 Geological Survey of Norway, Trondheim, Norway; 10 University of Alaska Anchorage, Department of Mechanical Engineering, Anchorage, AK USA; 11 NILU, The Fram Centre, Tromsø, Norway; 12 University of Regina, Johnson-Shoyama Graduate School of Public Policy, Regina, SK Canada; 13 Norwegian University of Science and Technology, Department of Interdisciplinary Studies of Culture, Faculty of Humanities, Trondheim, Norway; 14 Swedish University of Agricultural Sciences, Department of Crop Production Ecology, Umeå, Sweden; 15 Swedish University of Agricultural Sciences, Department of Plant Protection Biology, Lomma, Sweden; 16 Natural Resources Institute Finland, Maaninka, Finland; 17 The Arctic Six, Chair of Arctic Food Citizenship (2024-2026), Umeå University, Umeå Institute of Design, Umeå, Sweden; 18 The University of Southern Denmark, FoodLab, Department Business and Sustainability, Kolding, Denmark; 19 University of Iceland, Faculty of Civil and Environmental Engineering, Reykjavik, Iceland; 20 Agriculture and Agri-Food Canada, Ottawa Research and Development Centre, Ottawa, ON Canada; 21 University of Alaska Fairbanks, Department of Civil, Geological and Environmental Engineering, College of Engineering and Mines, Fairbanks, AK USA

**Keywords:** Environmental studies, Energy security, Agriculture, Decision making

## Abstract

Arctic food systems blend Traditional Ecological Knowledge with modern, often energy-intensive influences, triggered by colonization. Food systems’ future depends on alignment of tradition with innovation – at a pace determined by local communities.

## Teaser

Arctic food systems blend Traditional Ecological Knowledge with modern, often energy-intensive influences, triggered by colonization. Food systems’ future depends on alignment of tradition with innovation, facilitation of resilience and a heritage-driven interaction with the global economy – at a pace determined by local communities.

The need for secure and sustainable food on the one hand, and energy on the other hand, overlap in the circumpolar Arctic, for example in non-traditional land uses, such as modern greenhouse farming. This interdependency poses unique challenges, in a region that is a focus point for a range of global changes.

In the Arctic, climate change advances two to four times faster than the global average^1,2^, imposing exceptional stresses on the ecosystem. The high northern latitudes are also increasingly viewed as a strategically important region^3^. This perspective leads to significant resource allocations by respective states for development and infrastructure projects. Perceived opportunities for easier access to fossil resources such as oil and rare Earth metals on the one hand and Indigenous land rights on the other are important considerations to guide these developments. Furthermore, military presence and cooperation has significantly increased over the past 75 years^3^, in ways that have not necessarily been in support of embedded Indigenous communities, or respectful of their wishes.

Geographic isolation is a common feature of the Arctic that has long supported low-density human populations. As some, but not all, traditional Arctic rural communities may be in decline^3^, a steady increase in settler populations is triggering demographic and cultural shifts, with increases in a settlement-based lifestyle. Today, about 50% of the population in the Arctic are not Indigenous^4^. This further imperils the ways and cultures of diverse Arctic Indigenous nations, including food cultures^5^, whose political and cultural positioning remains marked by a history of colonialism. New, non-traditional land uses are often energy-intensive such as foods farmed^4^ where soil conditions and temperatures hinder farming. They also jeopardize the ways and cultures of diverse circumpolar Indigenous nations.

It is important to increase local capacity for producing food that meets local demand. One emerging constraint is access to sustainable, locally produced, and cost-effective energy: electrical power can be eight times as expensive in the Arctic as in lower latitude agricultural regions.

**Fig. 1:**
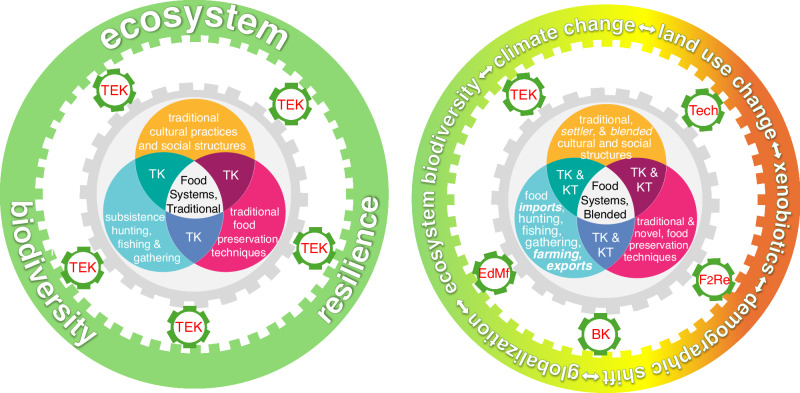
Shifting Arctic food systems. Traditional reliance on resilient ecosystem biodiversity (left) is shifting to integrated modern practices that blend tradition with technological advances to address rapid changes in ecosystem, demography and land-use (right). These shifts raise the question of how adaptation can be balanced with retaining cultural, social, and economic relevance and resilience. Traditional ecological knowledge (TEK); traditional knowledge (TK); knowledge transfer (KT); technology (Tech); fossil to renewable energy (F2Re); economic diversification and market forces (EdMf); blended knowledge (BK).

We argue that the Arctic is—partly by necessity, partly by a desire for economic diversification—primed for a revolution in food security and sovereignty that merges traditions with innovations to retain, revive, and reimagine local food production (Fig. 1).

## Complexity of Arctic food systems

The terms “Arctic” or “Arctic regions” may be variably understood^3^, as a reflection of geographic, cultural, ecosystemic, national, and economic boundaries. Internationally, it is the land north of the Arctic circle (66°30’ N latitude) or, in Canada, north of 60° N in latitude. Tundra, its defining biome, variably dips south of 60° N—for example, around Hudson Bay and Labrador in Canada—and rises above the Arctic circle in the Nordic countries and Asia. The characteristic permafrost soils that dominate the non-subglacial polar lands, occur sporadically and are isolated in sub-Arctic or non-Arctic alpine regions. These strict delineations do not necessarily align with cultural life or ecosystem features. In Arctic Indigenous Nations, continuity in cultural practices and food systems can extend further south into boreal biome lands.

Climate change in the Arctic leads to a range of changes: unpredictable weather patterns; an expansion of the active permafrost zone; extended periods of vegetation cover and increased summer ecosystem productivity^5^; shifting wildlife patterns^6^; more frequent and devastating forest fires^7^; destabilizing infrastructure^8^; and a decline in traditional food stocks^9^ have all been reported. The question is whether resilience can be built into food sustainability in the wake of these changes.

Infrastructure developments are important for the sustained development of the region. In northern Fennoscandia, transport infrastructure is well developed and integrated into national systems, whereas, in the Asian Arctic and parts of North America (outside the Alaskan/Alcan highway corridor), transport is often limited and challenged by significant distances. Infrastructure development is of major interest to regional cooperation initiatives that seek to foster economic and social advancements by strengthening transport corridors within the region and to external markets.

Traditional Arctic food systems, rooted in Indigenous cultures can be characterized by:

- Subsistence hunting, fishing, and gathering: Traditional hunting targets species, like seals, whales, caribou, and polar bears, are central to nutritional and cultural identity. Many communities rely on traditional foods such as fish (e.g., Arctic char, salmon, cod). Ice fishing is common during winter. Berries, seaweed, fungi, shellfish, and edible plants are gathered during the short Arctic summer.
- Traditional Food Preservation Techniques: Drying, smoking and fermenting methods help to preserve food for the long winter when fresh resources are scarce. Underground ice cellars and drums, crates or cavities within permafrost are traditionally used to store meat and fish.
- Traditional cultural practices and social structures, such as sharing food, are an important part of communal life; certain foods are associated with ceremonies and rituals.

These food systems are adapted to the harsh Arctic environment over centuries and generally sustainable. They rely on deep knowledge of the local ecosystem and careful management of resources, that is, traditional ecological knowledge^10^.

Access to traditional Arctic foods depends on access to traditional Arctic lands. Historically, the sustainability of traditional Arctic food systems is a fully integrated element of Arctic biodiversity. Rapid climatic changes disrupt hunting and gathering traditions, decoupling Traditional Ecological Knowledge-based social systems from drastically altered ecological realities. Biodiversity loss is often discussed but is neither effectively addressed when land-based food production is considered, nor are there locally verified monitoring mechanisms. Human activities or climate change that influences biodiversity alter traditional food practices, culturally sensitive foods, and approaches for food production, handling, and consumption, thus pushing communities further towards non-traditional foods, eventually challenging food sovereignty. Extensive pollution, “from soot to plastic, from methane to pesticides”^11^ including emerging and forever chemicals such as PFAS and mine effluents, leads to ecosystem-wide accumulation of xenobiotics that extends to traditional foods and can have drastic health impacts.

Declines in the utilization of and access to traditional food systems, and a shift to non-local foods are further entrenched by policies that are imposed by non-Indigenous governments. Often, such policies address food security concerns by facilitating food imports. This is well exemplified in governmental programs that address Arctic food security through increasing access to non-traditional foods: for example, the “[Canadian] Nutrition North Program, was introduced in 2011 to make healthy foods more accessible by providing food transportation subsidies to retailers”^12^. Consequently, a significant portion of the food supply in Arctic communities consists of non-traditional food, such as processed foods, canned goods or packaged snacks, that is expensive and tends to create nutritional imbalances.

Another consequence of government programs is the promotion of agricultural activities that are not traditional in the Arctic region, such as growing vegetables and herbs and providing fresh produce. Modern greenhouse techniques, hydroponics, and aquaponics are being explored, especially where soil and climate do not support land-based farming. These approaches perpetuate import-driven changes in dietary practices and solidify such shifts: food types that were non-local traditionally are now more and more being produced locally—at the cost of increased energy usage. Nevertheless, developing non-traditional food production without meaningful input from local communities is a road to failure^13^. What is technically possible and desirable for settler communities (e.g., the industry-driven Arctic Food Arena Project^14^) is not necessarily desirable for Indigenous communities^15^.

Importantly, some high value-added local foods show great export potential due to their “Arctic Origin”, which brings touted economic benefits to Arctic communities^4^. Often overlooked non-traditional food systems in the Arctic include large-scale commercial fishing, which, akin to resource extraction industries, might bring economic opportunities but compete with traditional subsistence activities.

## Blended Arctic food systems

There are growing movements in the Indigenous North, often within communities, that aim to both preserve traditional food practices and integrate beneficial aspects of modern food systems. While variably and increasingly heterogeneous, current Arctic food systems must, at the same time, target food sovereignty and address changing conditions, such as economic growth, rapid, climate-driven ecosystem changes, and food security. This can be accomplished by embracing locally adaptable innovations that optimize traditional food systems and increase local capacity for producing food that both meets local demand and satisfies markets for imported foods. People in the Arctic regions need food that utilizes local fuels for heat and production and is primarily cultivated, harvested, prepared, preserved, shared, sold, or traded within the boundaries of Indigenous respective territories, while allowing for additional ways to produce foods for export to regional and global markets.

Greenhouses can extend the growing season and provide fresh produce in harsh climates^16^. Permafrost thaw and loss of sea ice undermine traditional food storage methods, necessitating the increased use of energy-intensive refrigeration and transportation systems. However, the high cost of energy in the Arctic, predominantly derived from diesel generators, exacerbates food security challenges^17^.

## Conventional energy moves to green energy

Integrating renewable electricity generation, transmission, and non-disruptive thermal energy storage with food production offers the potential to achieve sustainable development of a food-energy nexus. Indigenous renewable energy initiatives are increasingly being adopted in the Arctic^18^:

- A combination of wind and solar energy sources is predicted to be a feasible, reliable, and affordable energy system in Longyearbyen, Svalbard^19^. In Iceland, geothermal water (for heating) and hydro- or geothermal-based power (for lighting) are used for greenhouse production.
- In Alaska and Canada, passive solar thermal greenhouse design^20^ and converting flare gas to heat, can extend the growing season for high-value produce crops as well as sequester carbon dioxide.
- Biowaste from local fisheries, slaughterhouses, sewage sludge and food waste can be converted into bioenergy^21^, with residual digestate and compost recycled into the circular economy.

Economic feasibility, environmental sustainability, availability of energy producers, and social acceptance for implementing mature renewable energy technologies for food production across Arctic regions must be systematically evaluated.

Practical limitations, such as limited windows to bring in renewable energy equipment, intensive engineering requirements due to permafrost, and heavy reliance on small-plane transportation, must be acknowledged. Nevertheless, prioritizing renewable energy investments, improving energy efficiency, and infrastructure for food storage and transportation enhance the sustainability of food systems while reducing both greenhouse gas emissions and financial burdens. Critically, these developments would provide communities with more control over their energy sources.

## Integration of Indigenous solutions with new technology

Complex ecosystem changes and evolving conditions in northern communities create the basis for a revitalization of traditional food systems that integrate sustainable harvesting of local flora and fauna with technology-driven sustainable practices for food production, storage, and transportation. At this point, it is important to ensure the resilience and self-sufficiency of Arctic communities.

Community-led co-management of resources is critical to building sustainability in the Arctic, maintaining food security and cultural identity amidst the pressures of globalization^22^; purposeful support from governmental and non-governmental organizations can help protect vital ecosystems and related food stocks. Integrating innovations is indispensable for addressing the challenges arising from unprecedented climate change. Collaborative approaches ensure that access to resources and economic development are balanced with the preservation of traditional livelihoods.

## Call to action

Arctic food systems blend traditions with modern influences, each with their strengths and challenges. Traditional food systems are rooted in the ecosystem, while non-traditional systems rely on technology-driven, energy-dependent food production and handling. With a precipitous new era of climate change, geo-political shifts, globalization and commodification of traditional foods, and demographic shifts, the traditional and modern approaches to Arctic food systems risk diverging. The speed of change might, even if inadvertently, favor policy support for technology over the ecosystem, targeting immediate needs rather than the long-term sustainability of site-adapted Arctic food systems.

An emerging paradigm must be acknowledged: social and policy movements targeting increased food security and sovereignty promote a return to traditional foods and the inclusion of introduced non-Arctic foods and food preservation techniques.

Sustainability challenges can be only resolved through region-specific, place-based social-ecological systems, linking ecosystem resilience and economic stability: the future of Arctic food systems will depend on how each Arctic community balances technological solutions with the preservation of cultural heritage.

Indigenous ownership, control, access, and possession over data collection and dissemination processes^23^, applying the FAIR and CARE principles^24^ is unquestionable.

Land-based education and capacity-building programs that incorporate both Indigenous knowledge and modern science are essential in creating self-determination among younger Indigenous generations with the skills needed to navigate the challenges of globalization while preserving their cultural heritage.

Regulatory frameworks, while acknowledging common challenges and the benefit of circumpolar integration of knowledge and resources, must recognize and facilitate effective implementation of place-based solutions.

We argue that the unique complexities that arise at the interface of extreme climate changes, social and cultural diversity^25^ and unique economic opportunities make the Arctic a model for swift adaptation to massive ecosystem changes. To master the challenges ahead, we need innovations that recognize, preserve and leverage diversity of local knowledge and cultural identities.
